# Digital twin enables radiosensitive organic speciation in 3D

**DOI:** 10.1126/sciadv.adw5444

**Published:** 2025-10-10

**Authors:** Laure Cazals, Agnès Desolneux, Simo Huotari, Lauren Dalecky, Christoph Sahle, Alessandro Mirone, Serge X. Cohen, Loïc Bertrand

**Affiliations:** ^1^ ENS Paris-Saclay, CNRS, PPSM, Université Paris-Saclay, 91190 Gif-sur-Yvette, France.; ^2^ ENS Paris-Saclay, CNRS, Centre Borelli, Université Paris-Saclay, 91190 Gif-sur-Yvette, France.; ^3^ Department of Physics, University of Helsinki, POB 64, FI-00014 Helsinki, Finland.; ^4^ ESRF, The European Synchrotron, CS 40220, 38043 Grenoble Cedex 9, France.; ^5^ CNRS, UVSQ, MNHN, Ministère de la Culture, IPANEMA, Université Paris-Saclay, 91192 Saint-Aubin, France.

## Abstract

State-of-the-art spectral imaging techniques using high-brilliance sources face an inherent trade-off between signal intensity and sample integrity, particularly in hyperspectral and multispectral imaging. Traditionally, optimizing acquisition parameters, such as source wavelength, intensity, and exposure time, relies on empirical adjustments to enhance image contrast. Here, we introduce a digital twin methodology to overcome these limitations. Focusing on x-ray Raman imaging, a powerful yet underutilized speciation probe constrained by low quantum efficiency, we demonstrate its application to sensitive organic samples. Our approach enabled a 10-fold reduction in acquisition time while maintaining operation below the damage threshold, paving the way for high-fidelity spectral imaging with minimal sample degradation.

## Teaser

Statistical optimization of spectral imaging allows the study of materials thought impossible to characterize.

## INTRODUCTION

Spectral imaging, which samples a spectrum at different points in space, has become a ubiquitous method for spatially classifying material systems (*1*). Hyperspectral images are collected over numerous bands that can be considered to provide a continuous spectrum at a (energy) resolution typically used for spectroscopy (*2*). In contrast, multispectral images are collected over a limited number of discrete (and usually wider) bands (*3*). A number of applied contributions compared the effectiveness of multispectral and hyperspectral imagery for different fields of applications, including satellite sensing, forest management, plant biology, food, and heritage chemistry (*4*–*7*). In chemical imaging, the large amount of information collected with both modalities implies long acquisition times, which can compromise a sample’s integrity (*8*–*10*). In particular, the high dose of electromagnetic radiation required to gather a sufficient signal can irreversibly damage inorganic and, even more so, organic samples (*11*, *12*). Different approaches are used to obtain sufficient count statistics while mitigating damage, such as cryogenic freezing (*13*, *14*). However, cold temperatures can themselves be very detrimental to the integrity of samples (*15*). Beyond these approaches, strategies based on increasing beam size, reducing beam flux and/or dwell time strongly impact the data collected. Dose reduction can pose the challenge of untangling the collected signal. A critical choice must be made to find a trade-off between sample preservation and the amount of knowledge collected.

When the probability of interaction between probe and matter is low, the choice of optimal parameters determines the success or failure of the experiment. Here, we consider a particularly promising speciation method, x-ray Raman spectral imaging. X-ray Raman inelastic spectroscopy, based on the energy loss excitation of core electrons by hard x-ray photons, is used for the in situ speciation of light elements (*16*–*18*). A fraction of the energy and momentum of incoming photons is transferred to electrons, leading to their excitation to an unoccupied state. These excitations allow scanning the binding energies that characterize atomic speciation (in particular chemical bonding, oxidation state and site symmetry) (*19*). The inelastic nature of this process makes it possible to characterize low-energy absorption edges at a few hundred electronvolts while probing and detecting with hard x-ray photons (typ. 5 to 20 keV). These can penetrate materials from a few tens of micrometers to several millimeters, depending on their composition, making x-ray Raman scattering (XRS) spectroscopy a unique bulk probe (*20*–*22*). Two-dimensional (2D) or 3D imaging can be done by scanning the surface while collecting signals in the depth dimension on the area detector (*23*). In practice, the probe’s unique ability to acquire the speciation of light elements in 3D has not led to the expected use. The very small effective cross section of inelastic x-ray scattering, several orders of magnitude smaller than that of the photoelectric effect, means that samples are subjected to extreme doses of radiation over a long acquisition time, beyond their damage threshold (*18*). Only a few examples of spectral imaging studies based on the method have been published to date, limited to specific samples that can withstand high levels of irradiation (*24*, *25*).

Here, we show that the development of a suitable digital twin unlocks the implementation of XRS imaging of radiosensitive organic samples. While, in practice, spectroscopists use the salient features of spectra as the main criterion to increase image contrast, we have modeled the influence of the main experimental parameters on the ability to factorize the generated datacube, taking into account the Poisson nature of the noise. A convex optimization approach toward sparser selection of spectral bands was designed. We show that this approach considerably improves the ability to recover sample chemistry. The hybridization of collected and synthetic data has made it possible to obtain 3D speciation of photosensitive materials. Our results show that using a large number of spectral bands within a limited time budget is not an optimal strategy. This work opens the way to faster and more efficient imaging modalities, preserving samples from biology to chemistry and material sciences.

## RESULTS

The quantity measured in a nonresonant inelastic x-ray scattering experiment, including XRS, is the double differential scattering cross section

$$\frac{d^{2}\sigma }{\mathrm{d\Omega}\;\mathrm{d\omega}}={\left(\frac{\mathrm{d\sigma}}{\mathrm{d\Omega}}\right)}_{\text{Th}}S\left(q,\omega \right)$$ (1)

Here, ${\left(\frac{\mathrm{d\sigma}}{\mathrm{d\Omega}}\right)}_{\text{Th}}$ is the Thomson scattering cross section, and S(q,ω) is the dynamic structure factor, which holds the information of interest

$$\begin{array}{c} S\left(q,\omega \right)=\sum_{f}|\langle f|\text{exp}\;\left(iq.r\right){|i\rangle |}^{2}\delta \;\left(E_{i}-E_{f}+\omega \right) \end{array}$$ (2)

where **ω** and **q** are the energy and momentum transferred to the system during the scattering process, respectively. ∣i〉 is the initial state, the ∣f〉s are the possible final states, and *E*_i_ (resp. *E*_f_) is the energy of the initial (resp. final) state. XRS results from scanning ω across an absorption edge of the target element. For small ∥q∥ values, XRS is formally equivalent to x-ray absorption spectroscopy (*26*) with sensitivity to the chemical environment of the absorbing/scattering atom. At larger momentum transfer XRS can provide even richer information through higher-order multipole contributions. XRS-based 3D imaging experiments rely on the capture of photons scattered along the beam using a pixelated surface detector, instantly forming a 1D image without having to solve inverse problems (direct tomography). A 4D volume is obtained by scanning the (y,z) surface and the energy range of interest (*23*, *27*).

We designed the digital twin of the detection of a 4D spectral image. Each acquisition scenario is integrated into the model and then evaluated in terms of its impact on image classification efficiency. We constructed an algorithm to identify the optimal scenario in terms of spectral band selection.

### Defining the acceptable absorbed dose

Probing matter with high-energy ionizing photons from several to tens of kiloelectronvolts induces radiation damage, mainly through two processes: the photoelectric effect and Compton scattering. The dose $D=E_{\text{dep}}/m$ is the deposited energy during irradiation, *E*_dep_, per sample mass, *m*. In the most common case of dose-dependent degradation pathways, *D* is proportional to the irradiation time at a given excitation energy *E* (*10*). For a homogeneous material, the attenuation of the beam typically follows a Victoreen law as a function of *E* outside the absorption edges of the constituent elements (*28*). Consequently, a small variation in *E* of a few tens of eV’s in a range of 5 to 20 keV, as in XRS, has very little impact on the dose. We can therefore translate the acceptable dose into a time budget per voxel, for a given experimental configuration and sample composition. In real life, experimenters play with two main sets of parameters: the number of energy points and the acquisition time per energy point. It is these parameters that we have decided to optimize.

### Constructing the ground truth of the spectral image of a radiation-sensitive sample

For a given sample position (y,z) and an ω energy loss, the pixelated detector collects a one-dimensional image of the beam path for each spherically bent crystal analyzer *a*. The intensity of single emitting points located along the beam is proportional to the dynamic structure factor S(q,ω,x), and **x** being the (x,y,z) coordinates of a voxel. As the effects of reabsorption in transmission can be neglected at high energy for organic samples, each voxel **x** of the sample is modeled as the sum of the emissions from a single point times the scatterers densities on the forward scattering analyzers for which the dipole approximation is valid (small **q**). We consider the generic case of a material leading to a 4D image, referred to here as a datacube, which can be modeled as a piecewise-constant spatial distribution of pure phases with noncollinear spectra. As this image is the result of a projection of scattered photons onto a voxel grid, partial volume effects, mixing of phase signals resulting from the finite spatial resolution of the detector, are taken into account at phase boundaries. Each phase is characterized by a phase spectrum $I_{k}^{0}$ with k∈⟦1,K⟧, and *K* being the total number of phases used to describe the sample. A linear mixture model is used to generate the ground truth, the ideal expected spectral image, taking into account the mathematical description of the sample morphology and partial volume effects. The ground-truth signal for each voxel x is modeled as

$$\begin{array}{c} I^{0}\left(x,e_{n}\right)=\sum_{k=1}^{K}p_{k}^{0}\;\left(x\right)\;I_{k}^{0}\;\left(e_{n}\right),\;\text{with}\;I_{k}^{0}=I_{k}^{\text{XRS}}+I_{k}^{\text{bkg}} \end{array}$$ (3)

with ${\left\{e_{n}\right\}}_{n=1,\ldots ,N}$ the energy points, *N* the total number of points in the spectral dimension, and $p_{k}^{0}\left(x\right)$ the factorization coefficients of each phase *k*. The $I_{k}^{0}$s are the sum of the signals collected by the crystal analyzers indexed by *a* at different forward scattering angles, corresponding to different low momentum transfers. They are written as the sum of the XRS signal $I_{k}^{\text{XRS}}$ and the background signal $I_{k}^{\text{bkg}}$, coming mainly from plasmons and electron-hole pair excitation.

### Simulating the acquisition time

As each pixel of the detector is a photon counter with a quantum efficiency of detection of approximately 0.85 for 12.9 keV, the only source of noise is shot noise (*29*). The acquisition time is simulated by the addition of synthetic shot noise (Poisson noise). The synthetic collected signal in **x** is modeled by a discrete random variable *Y* following a Poisson distribution with parameter λ, Y∼P(λ), thus having the expected value E(Y)=λ and variance Var(Y)=λ. For each energy point *e_n_* and each voxel **x** we simulate a Poisson distribution to model the signal collected on each crystal analyzer a∈⟦1,A⟧, *A* being the total number of crystal analyzers placed at different scattering angles

$$S^{a}\left(x,e_{n}\right)∼P\;\left(t\times \alpha^{a}\times I^{0,a}\;\left(x,e_{n}\right)\right)$$ (4)

with ${\left\{S^{a}\left(x,e_{n}\right)\right\}}_{n=1,\ldots ,N}$ being independent, and *t* the acquisition time per energy point directly linked to the number of photons counted on the detectors. The acquisition time is considered equal to the irradiation time, assuming that the beam does not irradiate the sample without data being acquired. The constant α*^a^* is referred to here as the detection efficiency, the product of (1 − air absorption) and the quantum efficiency (mainly due to angular acceptance) for a given incoming flux. The collected XRS image results from the combination of signals from several forward scattering detection angles with different α*^a^* values and therefore, different noise levels. To build this image, the signals from the crystal analyzers are interpolated onto common spatial and spectral grids, before being summed. Interpolation of these Poisson realizations preserves the Poissonian nature of the reconstructed image under the approximation in Supplementary Text. Assuming that the spectral information at low-momentum transfer is identical, the signals detected at different forward angles are statistically described as Poisson realizations of the same *I*^0^ spectrum. Equation 4 for all crystals combined becomes for each energy point *e_n_* and each voxel **x**

$$\begin{array}{c} S∼P\left(t\times \left(\alpha^{1}I^{0,1}+\cdots +\alpha^{A}I^{0,A}\right)\right)=P\left(t\;\times \;\alpha \;\times \;I^{0}\right)\;\text{with}\;\alpha =\sum_{a=1}^{A}\alpha^{a} \end{array}$$ (5)

### Factoring the synthetic datacube

To retrieve the phase distribution from the noisy image, we compare *S* to the mathematical model of the signal (Eq. 3). The least squares method is applied to estimate the factorization coefficients of phase spectra, $I_{k}^{0}$:

$$\begin{array}{c} \hat{p}\left(x\right)=\underset{p=\left(p_{1},\ldots ,p_{K}\right)}{\text{Argmin}}\sum_{n=1}^{N}{\left\{S\left(x,e_{n}\right)-t\alpha \;\left[p_{1}I_{1}^{0}\left(e_{n}\right)+p_{2}I_{2}^{0}\left(e_{n}\right)+\cdots +p_{K}I_{K}^{0}\left(e_{n}\right)\right]\right\}}^{2} \end{array}$$ (6)

It leads to the following linear equation

$$\hat{p}\left(x\right)=\frac{1}{t\alpha }{\left(G^{0}\right)}^{-1}{\left(I^{0}\right)}^{T}S\left(x\right)$$ (7)

with

$$\begin{array}{c} S\left(x\right)=\left[\begin{array}{c} S\left(x,e_{1}\right)\\ \vdots \\ S(x,e_{N})\; \end{array}\right]\;\text{and}\;I^{0}=\left[\begin{array}{c} \begin{array}{ccc} I_{1}^{0}\left(e_{1}\right) & \cdots & I_{K}^{0}\left(e_{1}\right) \end{array}\\ \begin{array}{ccc} \vdots \;\;\;\; & \ddots \;\;\; & \;\vdots \end{array}\\ \begin{array}{ccc} I_{1}^{0}\left(e_{N}\right) & \cdots & I_{K}^{0}(e_{N})\; \end{array} \end{array}\right] \end{array}$$ (8)

and $G^{0}={\left(I^{0}\right)}^{T}I^{0}$, the Gram matrix of the $I_{k}^{0}$s, a symmetric positive *K* × *K* matrix. The least squares estimator is unbiased for the calculation of factorization coefficients. The latter, defined in Eq. 7, gives the concentration of the phases present in the sample at voxel **x**, corresponding to the phase spectra. In addition, we apply a mean filter, taking advantage of the piecewise-constant nature of the ground truth. Underlying this method is the assumption that nearest-neighbor voxels are more likely to belong to the same class rather than the opposite and thus considers the spatial regularity affixed to the sample topology.

### Subsampling the set of energies

A critical choice must be made in the selection of the ${\left\{e_{n}\right\}}_{n=1,\ldots ,N}$ energy points, i.e., the definition of an energy grid, to maintain data interpretability. We define the criterion for choosing a subset of energy points as its ability to minimize the mean squared error of the estimated factorization coefficients $\hat{p}\left(x\right)$ at each voxel **x**. Calculating the latter leads to the following inequality (Supplementary Text)

$$\begin{array}{c} E\left[∥\hat{p}\left(x\right)-p^{0}\left(x\right){∥}^{2}\right]=\text{Tr}\left\{\text{Cov}[\hat{p}\;\left(x\right)]\right\}\leq \frac{C}{\alpha t}\times \text{Tr}\left[{\left(G^{0}\right)}^{-1}\right] \end{array}$$ (9)

with *C* a constant. At a fixed *t*, and for a number of energy points *M* < *N*, we define the following function to optimize the subsampling of the energy grid

$$F\left(\omega \right)=\text{Tr}\left[G^{0}{\left(\omega \right)}^{-1}\right]$$ (10)

where the **G**^0^ matrix has been rewritten to include the selection of energy subsets using the vector $\omega \in \Omega_{M}=\left\{\left(\omega_{1},\ldots ,\omega_{N}\right)\in {\left\{0,1\right\}}^{N}\right.\text{with}\left.\sum_{n=1}^{N}\omega_{n}\leq M\right\}$

$$G_{k,l}^{0}\;\left(\omega \right)=\sum_{n=1}^{N}\omega_{n}I_{k}^{0}\left(e_{n}\right)I_{l}^{0}\left(e_{n}\right)$$ (11)

with k,l∈⟦1,K⟧. At fixed *t* and *M*, the objective is to find the $\omega \in \Omega_{M}$ minimizing F(ω) leading to an optimal selection of energy points. We note that to discriminate *K* phases using least squares decomposition, a minimum of *M* = *K* energy points is required as a necessary condition for $G^{0}\left(\omega \right)$ to be invertible. Testing all possible $\omega \in \Omega_{M}$ energy subsets for each *M* < *N* requires long computation times. Consequently, we relax the problem by formulating it based on the Frank-Wolfe algorithm (*30*), where at each iteration, the problem is approximated linearly and minimized over a constrained convex region (details in Supplementary Text and fig. S1). We denote $\omega_{M}^{*}$ the deducted optimal energy grid.

### Assessing the quality of factorization of the synthetic datacube

For a given time budget *T* = *t* × *M*, we explore different acquisition scenarios. The noisy datacubes resulting from the numerical experiments are factorized. By assessing the quality of these factorizations, we sought to understand the best experimental strategy for a given *T*. To assess the factorization’s ability to recover **p**^0^ from $\hat{p}$, as a function of *M*, we apply a hard assignment to the calculated $\hat{p}$s to obtain a representation of the classes by the distribution of their maximum factorization coefficients. We then compare this classified image with the ground truth, excluding the boundary between classes and air. The success rate is calculated as the percentage of classified voxels belonging to the same classes as the corresponding ground-truth voxels.

### Collecting a 3D image at the carbon K edge of a paint sample

**Fig. 1.**
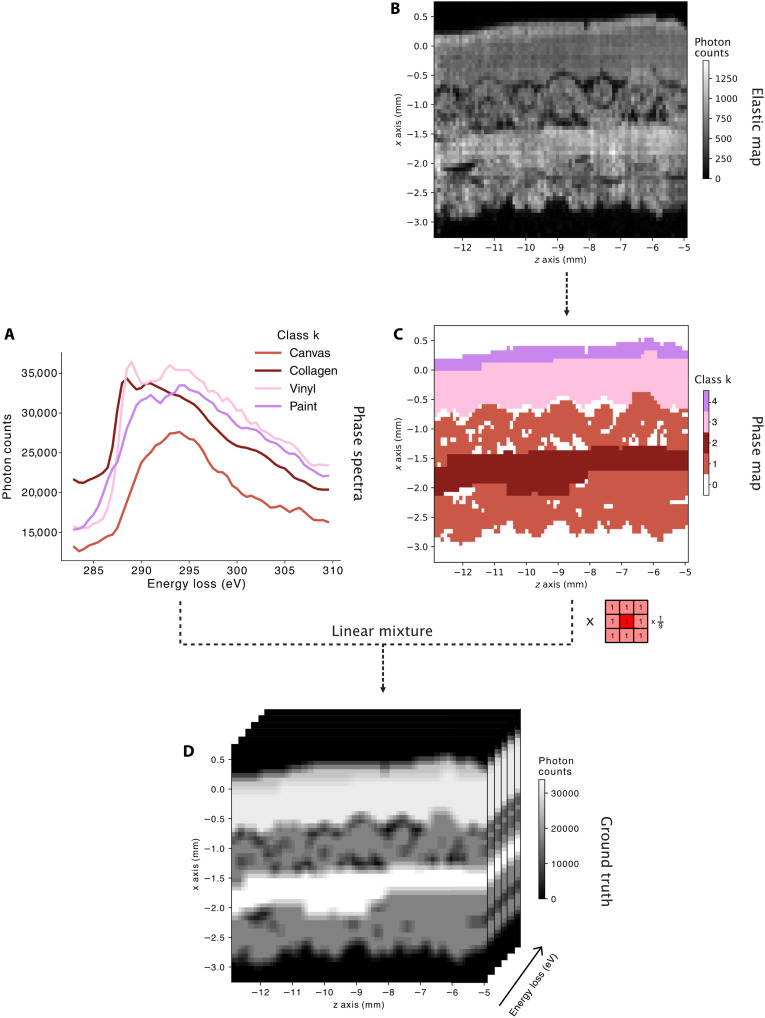
Construction of the ground truth of the spectral image of a radiosensitive sample. Experimental and numerical data are combined in three steps: (1) Phase spectra $I_{k}^{0}$ at the carbon K edge are obtained from raw materials (**A**). (2) The elastic signal (**B**) corrected for exponential attenuation effects is segmented into five activation images (**C**), generating a phase map without partial volume: 0 (air), 1 (canvas 1 and 2), 2 (collagen), 3 (vinyl), and 4 (paint). (3) A noise-free spectral volume is created by injecting the $I_{k}^{0}$s into the convoluted phase map $p_{k}^{0}\left(x\right)$, yielding the ground truth (**D**). For clarity, the image is displayed in 3D instead of 4D, with higher resolution along the *x* axis than in volume reconstruction.

**Fig. 2.**
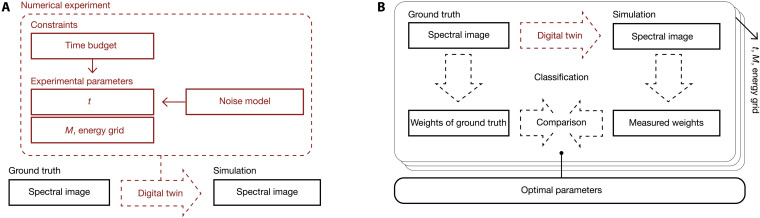
Schematic representation of the digital twin used in the simulation of experimental parameters and their optimization. (**A**) Digital twin, (**B**) optimization of experimental parameters.

**Fig. 3.**
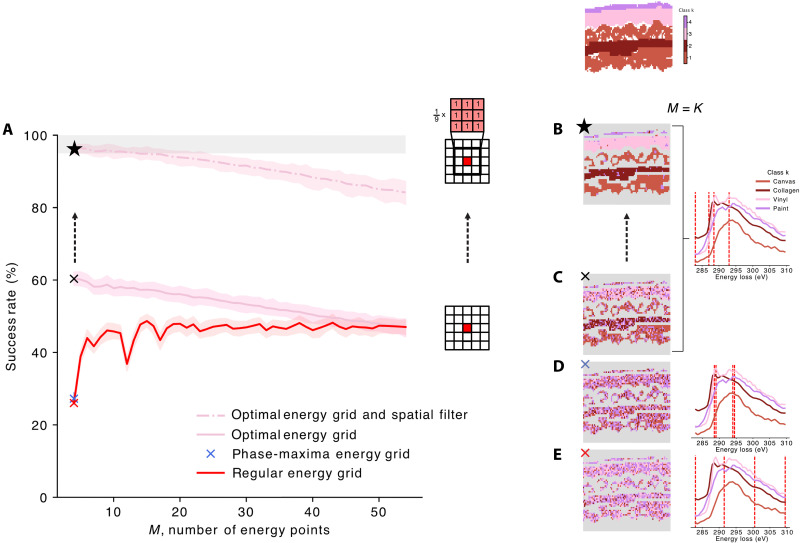
Optimization of experimental parameters within a time budget. (**A**) Success rate as a function of the number of energy points (*M*) for *T* = 1 s. Curves represent the average of 100 numerical experiments, with the associated 95% confidence interval. The region where the success rate exceeds 95% is highlighted in gray. (**B** to **E**) Selection scenario of energy point grids, where the number of energy points equals the number of phases (*M* = *K*): optimal energy grid [(B) and (C)], phase-maxima grid (D), and regular grid (E). Phase maps (left) and selected energies (right). The optimal energy grid map marked with a star results from mean spatial filtering. Gray areas corresponding to partial volume and air are not scored.

**Fig. 4.**
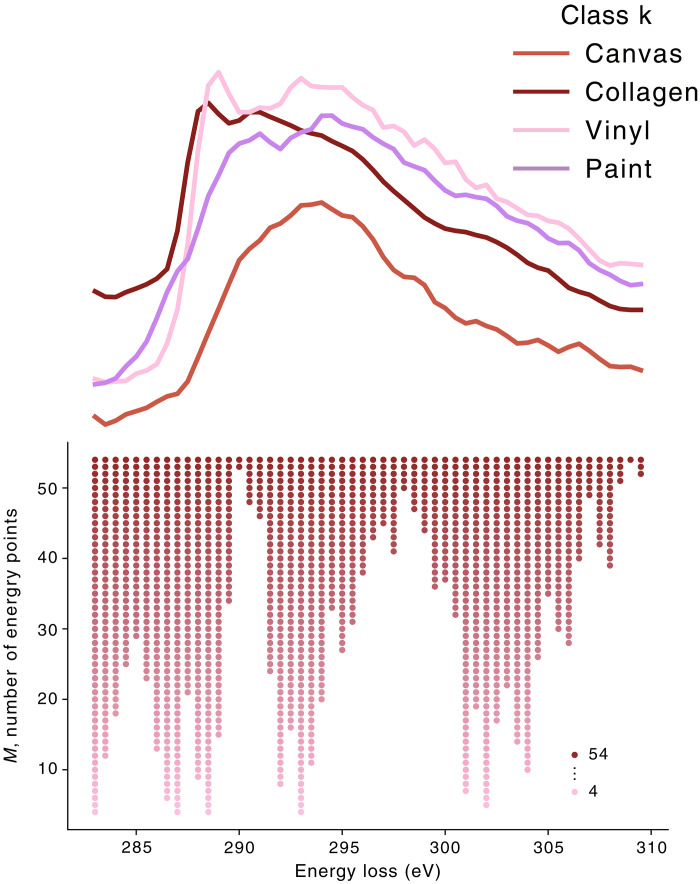
Optimal subsampling of the set of energies. Optimal energy grids obtained using the Frank-Wolfe algorithm with 1000 iterations for each *M* on the phase spectra obtained from raw materials. The optimal set of energy points, $\omega_{M}^{*}$, is highlighted by markers. Note that, here, once an energy point is selected, it is not discarded as *M* increases. The algorithm therefore behaves similarly to a greedy approach.

**Fig. 5.**
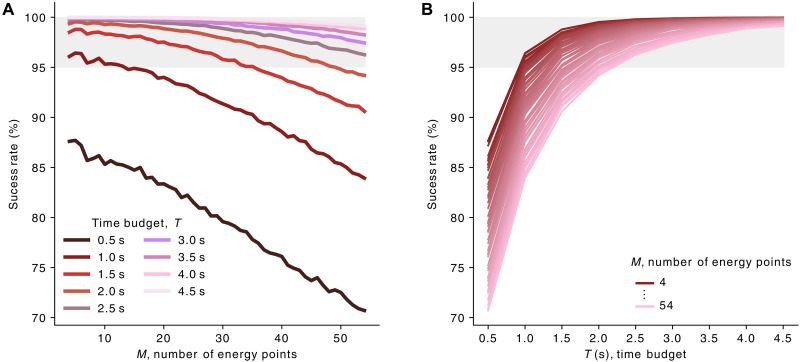
Evaluation of the success rate as a function of number of energy points and time budget. The success rate is plotted as a function of *M* (**A**) and *T* (**B**). Optimal energy grids and mean filtering were used as the optimal framework for phase recovery. The resulting curves are the average of 100 numerical experiments for each *M*. The region where the success rate exceeds 95% is highlighted in gray.

We selected a highly radiation-sensitive sample from studies of historical paintings (*31*). We adopted a historically accurate reconstruction procedure (*32*) to prepare a sample made of successive layers of canvas, rabbit-skin glue, vinyl glue, and paint (see Materials and methods). All experiments were performed at an estimated flux of 8 × 10^12^ ph/s at 199 mA. No physical change was observed on a large set of comparable samples with collection times below 4.5 s. We decided to add an extra margin for sample safety and set the time budget at *T* = 1 s. To design the ground truth of the spectral image: (i) we collected low-noise spectra of raw materials (canvas, collagen, vinyl, and paint) $I_{k}^{0}$, under cryogenic conditions at the carbon K edge (Fig. 1A and Supplementary Text); (ii) we collected the elastic signal from the sample (Fig. 1B) and classified it into five activation images (Fig. 1C); and (iii) we injected the $I_{k}^{0}$ into each respective activation image previously convolved at phase boundaries (Fig. 1D). The digital twin simulates the effects of (i) the number of energy points *M*, by subsampling the datacube, (ii) the acquisition time *t*, by applying a discrete Poisson random variable with parameter $t\times \alpha \times I_{k}^{0}$ (Fig. 2A). The α parameter was estimated experimentally (fig. S2). We applied the digital twin to a time budget *T* = 1 s for different *M* taking values between *K* = 4 and *N* = 54. Energy points were selected for each *M* according to regular energy grids traditionally used in hyperspectral mode. The projection onto the *N* energy points of the $I_{k}^{0}$s sometimes resulted in uneven subsampling (fig. S3). For each *M*, by simulating a noisy image at the corresponding *t*, so that *M* × *t* = *T*, and projecting onto the grids described above, we obtained a noisy synthetic image. These images were factorized using the least squares method, injecting the phase spectra $I_{k}^{0}$ (Fig. 1A), subsampled at *M* points on the corresponding energy grids. Classification efficiency was evaluated by comparing the success rate as a function of *M* (Fig. 2B). For small values of *M*, we observed an unstable behavior of the success rate (Fig. 3A). This was attributed to the fact that the grid lacks important spectral features for some *M*s, such as those at 288.5 and 289 eV (*M* = 7, 12, and 17). We then tested an experimental strategy commonly used by experimentalists working in multispectral mode, by selecting energy points corresponding to the maximum intensity of each pure phase spectrum (phase-maxima energy grid; Fig. 3D). The success rate of the phase-maxima energy grid at *M* = *K* is of the order of 27%, only 1% more than the corresponding regular grid (Fig. 3E). We then applied the optimal energy grids (Fig. 4). The following success rate is higher than for multispectral and hyperspectral selection modes, up to double for *M* = *K* (Fig. 3C). To assess the optimization of energy point selection for phase spectra, the upper limit Eq. 9 at ω^*^ was compared with numerical experiments (fig. S4). We applied a mean filter to the factorization coefficients obtained after projection onto the optimal energy grids. This denoising method increased the success rate by 35% (Fig. 3B). In a final step, we varied the time budget *T*, simulating *N* − *K* collection conditions by adjusting *M* so that *T* = *t* × *M* with T≤4.5 s. The parameter *M* took values between *K* = 4 and *N* = 54 for each *T*, each time using the corresponding optimal energy grid (Fig. 4), and by applying least squares upstream of mean filtering. The success rate increases with *T*, as expected (Fig. 5). We systematically observed a similar trend to that with *T* = 1 s: The success rate increases as the number of energy points decreases and, consequently, as the acquisition time per point increases. The difference in success rate between a few energy points and a large number of energy points decreases as *T* increases, highlighting the fact that for large time budgets, an optimal choice of *T* distribution between *t* and *M* does not notably alter phase recovery, whereas for low *T* values, this optimization can be a game changer. By arbitrarily setting the acceptable success rate at 95%, we can design an experiment for a time budget set at 1 s without having to reach the safety limit, provided we work in multispectral mode with an optimal energy grid and mean filtering.

## DISCUSSION

The digital twin–based approach proves to be a powerful tool for quantitatively intercomparing experimental strategies. For a constant time budget of between 0.5 and 4.5 s, the success rate obtained increases with time per energy point (and therefore inversely with the number of energy points). This indicates that collecting whole spectra made up of a very large number of spectral bands (hyperspectral strategy) is not an optimal choice for segmenting the image, and that it is more efficient to rely on longer exposure at a smaller number of energies (multispectral strategy). With a time budget of 4.5 s, the choice between these two modalities has an impact limited to a few percent on the success rate. However, for low time budgets, limiting the number of energy points has a considerable impact on the success rate, leading to an increase of over 25%. In short, multispectral strategy becomes increasingly essential as the signal-to-noise ratio diminishes. In particular, it enabled 3D imaging of carbon speciation in a paint sample for *T* = 1 s. The method described here requires complete knowledge of the sample phase composition. Further development could incorporate the possibility of unknown or unanticipated phases, requiring acquisition at a greater number of energy points via dynamic bidirectional updates during experimental run.

The term “digital twin” has various meanings across and within scientific fields. Here, we refer to a virtual model of a real experimental process, generated by combining experimental and synthetic data, capable of simulating results to prescribe actions by numerically varying parameters across a space inaccessible to physical testing. The realistic noise model developed links the simulation and practical acquisition parameters. We can thus identify avenues for the development of future expert software to optimize experimental protocols. A certain degree of robustness would be necessary, as the results are highly dependent on the topology of the sample and the chemistry of the constituent phases. Several types of combination are conceivable, as the topology and phase spectra of the constituent phases can either be experimental or synthetic. For example, several strategies can be used to assign the $I_{k}^{0}$s: (i) electron energy loss spectra of gas-phase molecules can be used or linearly combined (*33*); (ii) XRS signals can be simulated using software such as ERKALE (*34*), FEFF (*35*), FDMNES (*36*), StoBe-deMon (*37*, *38*), or Quantum ESPRESSO (*39*–*41*), ideally adding the Compton contribution (*27*); and (iii) XRS spectra can be collected directly on reference materials with denoising to retain only the Compton and Gaussian signals after spectral decomposition, or using cryogenic conditions as done here. Similar simulations exist for other probe-matter interactions. Beyond the digital twin framework, by considering a sample sketch for the spatial map, a model requiring no real experiments is conceivable to define the sample topology.

In this work, our numerical experiments enabled us to define the optimum experimental parameters for a given time budget set at a maximum of *T* = 4.5 s, defined in terms of radiation damage threshold for a given flux. We have observed that classification for such a budget is satisfactory. Acquisition could even be performed at a lower signal-to-noise ratio. In recent years, numerous studies have shown that the first particle to interact with a given sample may already be a source of alteration. This calls into question the nature of the information gathered on certain systems that are only known through the delivery of high doses. What kind of knowledge could we obtain about these samples by working with just a few particles? In line with the ALARA (as low as reasonably achievable) concept of risk management, we could consider the shortest irradiation time to reach a classification threshold, to keep the radiation dose as low as possible. We believe that digital twin methods, such as the one proposed here, could open up a range of possibilities in the study of matter by irradiation, and not just photosensitive matter.

The development of x-ray imaging, made possible by the advances in efficient imaging detectors and micro- and nanofocusing optics from the 1990s onward, has made it possible to produce hyper- and multispectral images, giving rise to a sometimes heated debate between spectroscopists and imaging specialists on the advantages of the different approaches. Our result shows that maximizing the number of energy points (“collecting whole spectra”) may be a less successful strategy for segmenting an image than collecting optimal images at well-defined energies, not necessarily those that an instrumentalist would have selected in the first place. We show that a digital twin approach enables 3D speciation of a paint sample at the carbon K edge, which was previously considered challenging, if not impossible. This optimization strategy therefore opens new avenues for data collection on radiation-sensitive systems. While we show that acquisition parameters can be optimized during experimental planning, assuming knowledge of the constitutive phases, our work envisions a future where a digital twin–driven machine agent could dynamically guide the evolution of acquisition parameters in real time during data acquisition.

## MATERIALS AND METHODS

All XRS imaging and spectral data were collected on the ID20 beamline of the European Synchrotron Radiation Facility using the direct tomography configuration (*23*, *42*) at a ring current of 200 mA. The incident photon beam energy was set at 12.9 keV with an energy resolution of 1.2 eV using a liquid nitrogen–cooled Si(111) double-crystal monochromator, coupled to a Si(311) postmonochromator. The beam is focused by a set of Kirkpatrick-Baez mirrors to a spot size of 12.5 μm by 12.5 μm (*V* × *H*) for the raw materials and 21 μm by 28 μm for the painting sample at the sample position. It passes through a (y,z) point, and the scattered photons are then captured by spherically curved analyzer crystals in Johann geometry, which act as precise energy filters. The signal from the beam path through the sample is reflected by each crystal and collected by a 2D photon-counting detector with a pixel size of 55 μm, giving the third spatial dimension *x*. 2D (x,z) volume of the painting sample was collected by scanning the x-ray beam vertically (*z* axis) over the sample surface at incident energy to target zero energy losses. Steps of 10 μm in vertical (stz) was used.

We used the IXStools library to convert the raw signals detected into a 2D volume for imaging and 1D for raw material spectra. The signals on the six modules are separated into 72 regions of interest (ROIs) corresponding to the 72 crystals. Each ROI is interpolated on the excitation energy grid and, for imaging, on a common *x* axis with steps of 0.2 mm using nearest-neighbor interpolation. The raw materials were studied under cryogenic conditions (Supplementary Text).

### Paint layers

We made a historically accurate reconstruction reproducing the pictorial practice of a major contemporary postwar artist: Simon Hantaï (1922–2008). It was prepared by first applying multiple layers of rabbit skin glue (Laverdure) to a linen canvas (no.811, Sennelier). For each layer, the rabbit skin glue was left to dry overnight for full evaporation of water. Next, a second linen canvas (no.811, Sennelier) was glued onto the rabbit skin glue layer using a small amount of fish glue and rice starch mixture. Once dry, a thick layer of vinyl glue (Caparol) was applied, and left to dry overnight. A mixture of acrylic binder (Laverdure) and Napthol Crimson organic pigment (Sennelier) was applied. Each material making up the stratigraphy was isolated and prepared for the collection of phase spectra (Supplementary Text).
