# Supplementary material for: Digital twin enables radiosensitive organic speciation in 3D
Source: Sci Adv. 2025 Oct 10;11(41):eadw5444. doi: 10.1126/sciadv.adw5444 (PMC13155532; doi:10.1126/sciadv.adw5444)
Supplement: Supplementary file 1 — Supplementary Text Figs. S1 to S4 [file sciadv.adw5444_sm.pdf]

Science Advances
AAAS

Supplementary Materials for

Digital twin enables radiosensitive organic speciation in 3D

Laure Cazals *et al.*

Corresponding author: Loïc Bertrand, loic.bertrand@ens-paris-saclay.fr

Sci. Adv. **11**, eadw5444 (2025)
DOI: 10.1126/sciadv.adw5444

This PDF file includes:

Supplementary Text
Figs. S1 to S4

Supplementary Text

Detailed Materials and Methods

Cryogenic set-up A 3D printed *cooling* chamber was designed at ID20 with Kapton windows on the beam path. A liquid nitrogen tank supplies cold N_2 gas inside the chamber, enabling a temperature of 173 K to be reached close to the sample position. The system is controlled by the Eurotherm nanodac 220 V recorder/controller. The cooling chamber is coupled to a fast shutter based on a chopper mechanism, allowing the sample to be irradiated only when data is acquired.

Raw materials The raw materials used in the digital twin were pressed into 6 mm-diameter pellets and used as references to generate the phase spectra. By cutting linen canvas (n°811, Sennelier) into pieces and pressing the resulting powder, we produced our *canvas* reference. Reference *collagen* was produced by pressing rabbit skin glue purchased in granulated form from Laverdure. We applied a thick layer of vinyl glue (Caparol) and let it dry before cutting it into pieces and pressing it to obtain *vinyl*. Finally, by mixing quinacridone red with acrylic binder (0.05–0.95) and letting it dry, we obtained a pictorial layer, which we then transformed into a pellet to obtain *paint*.

Spectral acquisition on raw materials Spectra of selected raw materials were collected using XRS spectroscopy in cryogenic conditions, by scanning the energy loss between 270 eV and 320 eV (rate: 1 s per point, several acquisitions, Si(311) post-monochromator, energy resolution: approx. 1.2 eV). The flux at 12.9 keV was estimated at 8×10^{12} ph/s at 199 mA. To generate the phase spectra I_k^0, the signals were integrated on 48 forward-scattering analyzers at angles of $2\theta = 32.2°$ and 45° using nearest-neighbor interpolation, and smoothed using convolution with an aperture window of 1 eV, slightly less than the post-monochromator energy resolution. During acquisition, the incident flux dropped by a factor of five, so we corrected the signal intensity accordingly. We corrected the spectra for each material by their number of measurements summed together.

X-ray Raman imaging

High-flux, high-brightness monochromatic synchrotron sources favored the use of X-ray Raman inelastic spectroscopy (*22,20,21*). Over the last ten years, innovative XRS-based imaging strategies

have emerged (*27, 43, 42, 44*). In particular, Huotari et al. developed a direct tomography approach based on the point-to-point focusing of spherically bent analyzer crystals in the Johann geometry. Photons scattered along the beam are captured by a pixelated area detector, instantly forming a 1D image without having to solve inverse problems. From there, it is possible to move on to 2D or 3D imaging by scanning the surface (y and z axis) while collecting signals in the depth dimension (x axis) on the area detector. This method has been used for a limited number of applications, such as the description of X-ray-induced chemical reactions (*45*) or for 3D speciation in radiation-hard samples, such as meteorites (*25*) or organic fossils (*24*).

In practice, for each crystal analyzer a, the signal is collected on a 2D pixel detector for each (y, z) scan point. Then, by summing the signal over the beam width, one-dimensional x_a axes are recovered. These have a different pixel resolution, depending on how the beam is projected onto the detector. In addition, the 72 crystal analyzers collect data at different energy grids. To reconstruct a 2D spectral image for each point (y, z) summarizing the contribution of several crystal analyzers, the data are projected onto the excitation energy grid and onto an x axis using `IXStools` software (formerly `XRStools`) (*43, 46*). In this paper, we consider the nearest-neighbor interpolation for these last two dimensions to preserve the Poisson statistics in the reconstructed image. We note that the detectors' energy grids retain the energy resolution of the excitation energy grid, so that each point on the latter benefits from a contribution from each crystal analyzer, except at the boundaries, making the spectral information on each reconstructed pixel physically meaningful. In addition, we choose the reconstruction configuration where at least one pixel per analyzer contributes to the reconstructed pixel, which results in a loss of spatial resolution, but avoids having a zero contribution from certain detectors. In the noise model, we place ourselves in the least favorable scenario, in which only one pixel from each crystal contributes to the reconstructed pixel. In doing so, we overestimate the noise in the digital twin compared with a real experimental sample, thus preserving the validity of the results.

A stringent current limitation of the use and development of XRS imaging is the very low effective cross-section of inelastic x-ray scattering. It leads to the detection of a very small number of photons, compared with X-ray absorption spectroscopy, when working with the same acquisition times. Various strategies can be used to compensate for this shot noise arising from the counting statistics, starting with the selection of regions of interest (ROIs) on the detectors. In doing so, the

signal-to-noise ratio is often considerably improved by eliminating the contribution of background noise before integrating over the beam width to obtain the depth axis, known as the x axis. Once the ROIs have been selected on several detectors at different angles, the signals can be interpolated on the same energy and x axis grid and summed, provided they have the same momentum, and therefore the same spectral behavior. In this way, the number of photons per pixel is significantly increased. However, even with these strategies and working with long acquisition times, down to a few seconds or tens of seconds, the number of photons remains very low.

Subsampling the set of energies

Covariance calculation Solving the linear equation derived from the least-squares method, we have:

$$\hat{\mathbf{p}}(\mathbf{x}) = \frac{1}{t\alpha}(\mathbf{G}^0)^{-1}(\mathbf{I}^0)^{\mathrm{T}}\mathbf{S}(\mathbf{x}) \tag{S1}$$

We deduce the expected value:

$$\mathbb{E}[\hat{\mathbf{p}}(\mathbf{x})] = \frac{1}{t\alpha}(\mathbf{G}^0)^{-1}(\mathbf{I}^0)^{\mathrm{T}}\mathbb{E}[\mathbf{S}(\mathbf{x})] = \mathbf{p}^0(\mathbf{x}) \text{ since } \mathbb{E}[\mathbf{S}(\mathbf{x})] = t\alpha\mathbf{I}^0\mathbf{p}^0(\mathbf{x}) \tag{S2}$$

The least-squares estimator is therefore unbiased for a signal with a Poisson distribution. Since $\mathbf{G}^0$ is symmetric, the covariance is written:

$$\mathrm{Cov}(\hat{\mathbf{p}}(\mathbf{x})) = \frac{1}{t^2\alpha^2}(\mathbf{G}^0)^{-1}(\mathbf{I}^0)^{\mathrm{T}}\mathrm{Cov}(\mathbf{S}(\mathbf{x}))\mathbf{I}^0(\mathbf{G}^0)^{-1} \tag{S3}$$

with:

$$\mathrm{Cov}(\mathbf{S}(\mathbf{x})) = \mathrm{Diag}(t\alpha\mathbf{I}^0(\mathbf{x}, e_n)) \tag{S4}$$

as $\{S(\mathbf{x}, e_n) \sim \mathcal{P}(t \times \alpha \times I^0(\mathbf{x}, e_n))\}_{n=1,\dots,N}$ are independent. Since $\mathrm{Cov}(\mathbf{S}(\mathbf{x})) \leqslant t\alpha C\mathbb{I}_N$, as an inequality between positive symmetric matrices, with $C = \max_{\mathbf{x}, e_n} \mathbf{I}^0(\mathbf{x}, e_n)$ a constant, we can rewrite:

$$\mathrm{Cov}(\hat{\mathbf{p}}) \leqslant \frac{C}{\alpha t} \times (\mathbf{G}^0)^{-1} \tag{S5}$$

Frank–Wolfe algorithm The mean squared error of the estimated factorization coefficients $\hat{\mathbf{p}}(\mathbf{x})$ at each voxel $\mathbf{x}$ is:

$$\mathbb{E}\left(\|\hat{\mathbf{p}}(\mathbf{x}) - \mathbf{p}^0(\mathbf{x})\|^2\right) = \mathrm{Tr}\left(\mathrm{Cov}\left(\hat{\mathbf{p}}\left(\mathbf{x}\right)\right)\right) \leq \frac{C}{\alpha t} \times \mathrm{Tr}\left((\mathbf{G}^0)^{-1}\right) \tag{S6}$$

with C a constant. At a fixed t, and for a number of energy points $M < N$, minimization of the convex function $\mathrm{F}(\omega) = \mathrm{Tr}(\mathbf{G}^0(\omega)^{-1})$ is performed on the convex set $\omega \in \Omega'_M = \{(\omega_1, \dots, \omega_N) \in [0,1]^N \text{ with } \sum_{n=1}^{N} \omega_n \leq M\}$. At each step J of the Frank–Wolfe algorithm , we minimize the linear approximation of the problem over the constrained convex region Ω'_M by taking the M smallest values of $\nabla\mathrm{F}$ (figure S1).

```
Input: M, J_max

Initialization ω := {M/N, ..., M/N}

while J < J_max do
    Compute the gradient ∇F(ω)
    Sort the values of the gradient
    Keep the M smallest values and record them in z
    Update ω ← ω + 2/(J+M) (z − ω)
    Update J ← J + 1
end while

Sort ω

Return the indices of the M largest values
```

Figure S1: **Frank–Wolfe algorithm.** Algorithm used to calculate the optimal energy grid for each M.

To apply the Frank–Wolfe algorithm, we need to have an explicit formula of $\nabla\mathrm{F}(\omega) = \frac{\partial \mathrm{Tr}(\mathbf{G}^0(\omega)^{-1})}{\partial \omega}$. As $\mathbf{G}^0(\omega)\mathbf{G}^0(\omega)^{-1} = \mathbb{I}_N$, we have:

$$\frac{\partial \mathbf{G}^0(\omega)}{\partial \omega_n}\mathbf{G}^0(\omega)^{-1} + \mathbf{G}^0(\omega)\frac{\partial \mathbf{G}^0(\omega)^{-1}}{\partial \omega_n} = 0 \Longrightarrow \frac{\partial \mathrm{F}(\omega)}{\partial \omega_n} = -\mathrm{Tr}(\mathbf{G}^0(\omega)^{-1}\frac{\partial \mathbf{G}^0(\omega)}{\partial \omega_n}\mathbf{G}^0(\omega)^{-1}) \tag{S7}$$

The matrix $\mathbf{G}^0(\omega)$ has the following formula:

$$\mathbf{G}^0(\omega) = \begin{pmatrix} \sum_n \omega_n I_1^0(e_n)^2 & \cdots & \sum_n \omega_n I_1^0(e_n) I_K^0(e_n) \\ \vdots & \ddots & \vdots \\ \sum_n \omega_n I_K^0(e_n) I_1^0(e_n) & \dots & \sum_n \omega_n I_K^0(e_n)^2 \end{pmatrix} \tag{S8}$$

and its partial derivative:

$$\frac{\partial \mathbf{G}^0(\omega)}{\partial \omega_n} = \begin{pmatrix} I_1^0(e_n)^2 & \cdots & I_1^0(e_n) I_K^0(e_n) \\ \vdots & \ddots & \vdots \\ I_K^0(e_n) I_1^0(e_n) & \dots & I_K^0(e_n)^2 \end{pmatrix} \tag{S9}$$

With a similar calculation we obtained $\nabla^2 \mathrm{F}(\omega)$ and demonstrated its positivity, proving the convexity of $\mathrm{F}(\omega)$.

3D image at the carbon K edge of a paint sample

The paint sample was used to acquire the spatial distribution of phases and estimate T. The spectral image of the paint sample was described by five activation images corresponding to their constituent materials: *canvas*, *collagen*, *vinyl*, *paint* and *air*.

Establishing the time budget The time budget for preserving the chemical and morphological integrity of paint fragments is difficult to determine because of their heterogeneity. In this study, it was defined by visual inspection after irradiation. The acquisition of an image composed of spectra collected for 4.5 seconds each (per pixel) or less, using a high-resolution configuration at 12.9 keV with a circulating electron current of 200 mA at the beamline ID20 (ESRF), did not result in any noticeable darkening or spatial expansion of similar samples.

Constructing the ground truth We collected phase spectra of selected raw materials using XRS spectroscopy in cryogenic conditions at the carbon K edge. To generate the phase spectra I_k^0, the signals were integrated on 48 forward-scattering analyzers using nearest-neighbor interpolation, and smoothed using convolution with an aperture window of 1 eV, slightly less than the post-monochromator energy resolution. For the *air* phase, we set $I_{\mathrm{air}}^0 = 0$ for all $n \in [[1, N]]$. In parallel, the paint sample was imaged at the elastic line using a single crystal analyzer from the module oriented at an angle 121°. The signal was corrected for attenuation effects, then segmented on the basis of visual identification of intensity and shape differences between the layers forming the stratigraphy, resulting in five activation images. We performed optical convolution to simulate the mixing resulting from partial volume effects. We ended up with $K = 4$ chemical phases, each defined by a I_k^0 spectrum and a spatial distribution. The I_k^0 of the raw materials have been injected into the factorization coefficient map to form the ground truth. This models the data collection on 48 crystal analyzers in forward scattering at a long acquisition time per energy point collected with a Si(311) post-monochromator with a circulating electron current of 200 mA.

Simulating the acquisition time We generated the synthetic datacube using a Poisson discrete random variable with parameter $t \times \sum_{a=1}^{A} \alpha^a \times I_k^0$. Parameters α^a were first estimated from real data collected twice at 1 s per point on the raw rabbit skin glue used for the *collagen* spectrum. For each analyzer a in forward scattering, we summed the data over the beam width dimension to obtain a signal for each pixel in x axis, the depth dimension. Placing ourselves in the worst-case scenario described in *X-ray Raman imaging*, we considered each of these pixels to be equivalent to those in the reconstructed volume. Neglecting reabsorption, we took as the expected value of the signal per analyzer the average value of pixels in the x axis. Dividing the average spectra for each a by the phase spectrum *collagen*, we obtained α_a. Then, we added them together and obtained α as a function of energy points. We divided by two (the number of acquisitions) and took the median value on the energy dimension. We have thus reached the value α for 1 s (figure S2), and then, by simply varying t, we are able to model the corresponding noise on the ground truth image.

Subsampling the set of energies Three sets of energy grids were assessed using the digital twin: (1) the regular grids (figure S3), (2) the phase-maxima energy grid (Figure 3D), (3) the optimal energy grids (Figure 4).

Comparing theory and numerical experience Using the phase spectra taken from the raw materials, we have plotted the function $\mathrm{F}(\omega_M^*)$ as a function of M. As expected, a decrease is observed when M increases. Multiplying $\mathrm{F}(\omega_M^*)$ by $\frac{C}{\alpha t} = \frac{M \times C}{\alpha T}$, we obtained the optimized upper limit of the sum of the variances of $\hat{p}_k$ as a function of M. To evaluate this curve, we have plotted the corresponding experimental sum variance on the factorization coefficients obtained from 100 numerical experiments at ω_M^*, and we observed a similar trend (figure S4).

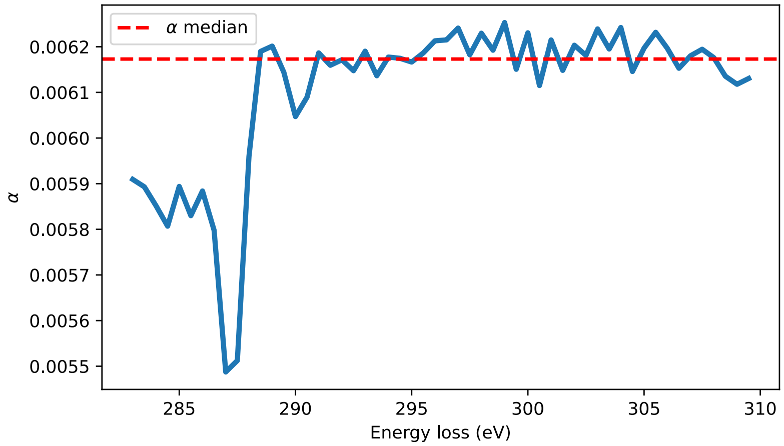

Figure S2: **Determination of the detection efficiency α.** Sum of α^a as a function of energy points and its median.

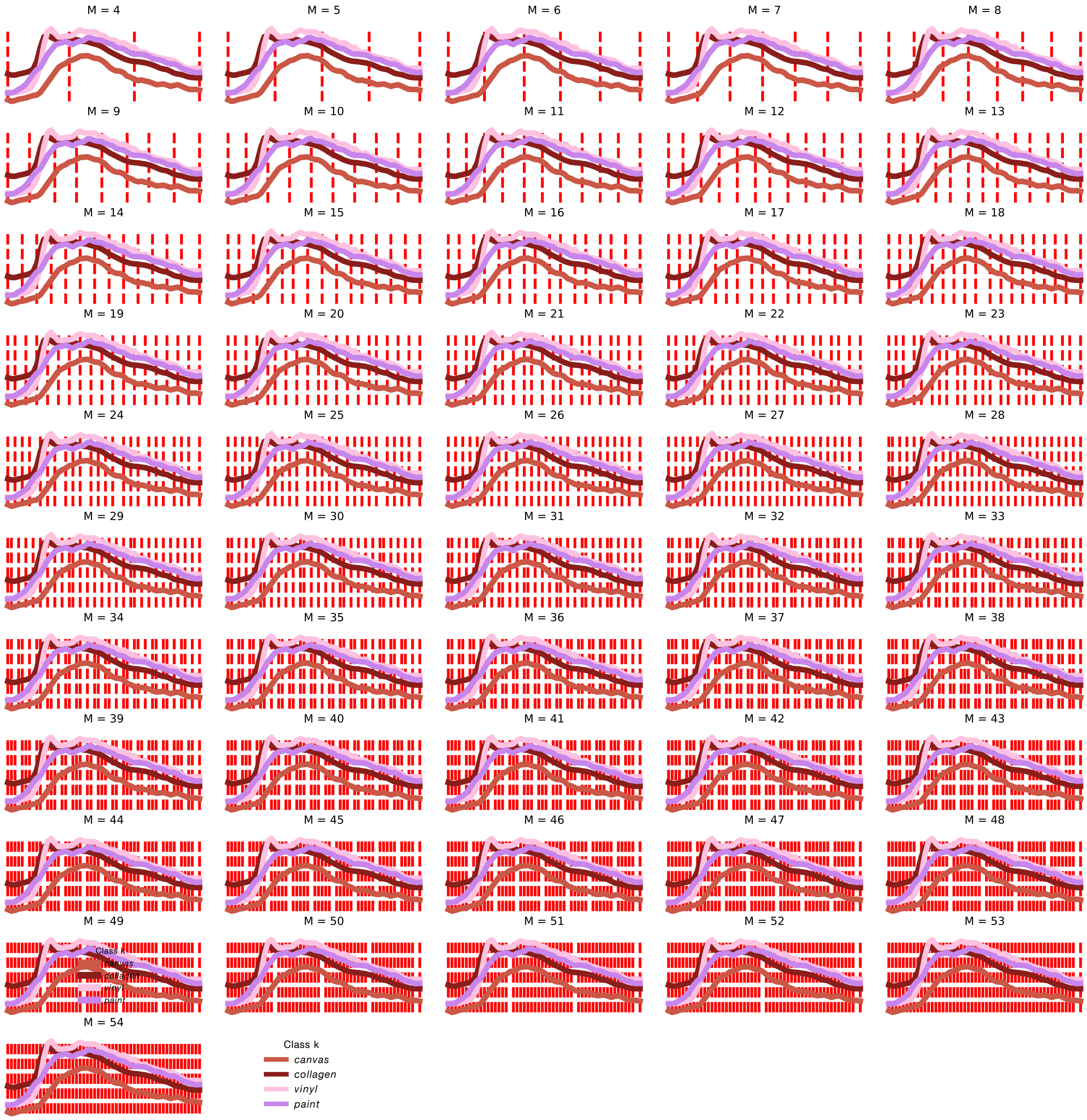

Figure S3: Regular energy grids for different values of M.

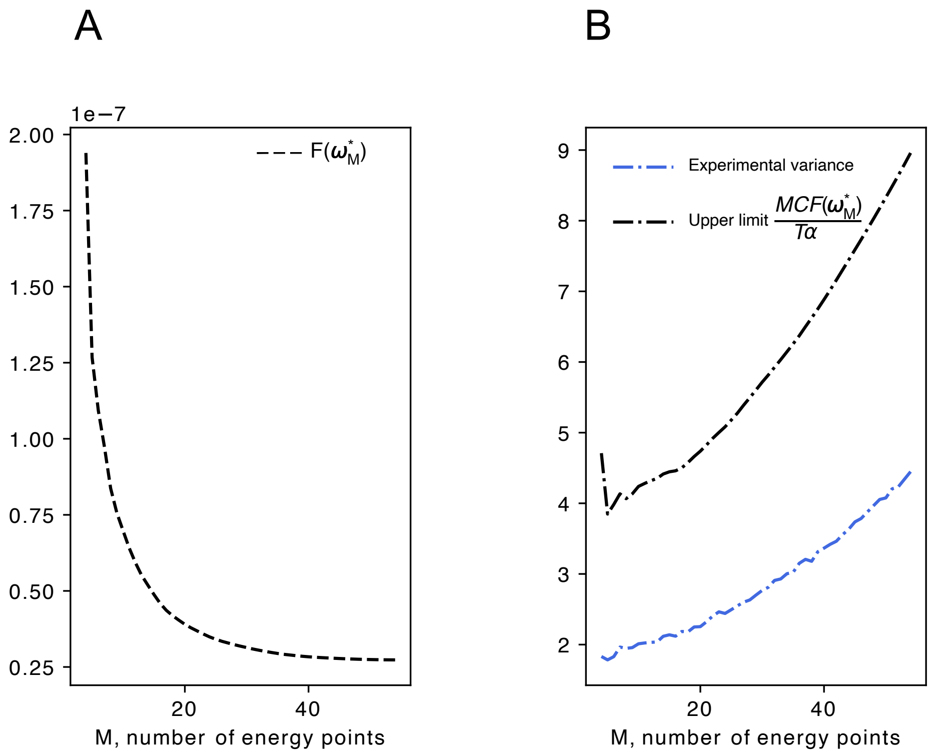

Figure S4: **Comparison of theory and numerical experiments.** (A) $\mathrm{F}(\omega_M^*)$ as a function of M for the raw materials spectra. (B) Experimental sum of the variance $\hat{p}_k$ over 100 numerical experiments and its theoretical upper bound as a function of M.
